# Untargeted Metabolomic Identifies Potential Seasonal Biomarkers of Semen Quality in Duroc Boars

**DOI:** 10.3390/biology14080995

**Published:** 2025-08-04

**Authors:** Notsile H. Dlamini, Serge L. Kameni, Jean M. Feugang

**Affiliations:** Department of Animal and Dairy Sciences, Mississippi State University, Starkville, MS 39759, USA; nhd30@msstate.edu (N.H.D.); sk2086@msstate.edu (S.L.K.)

**Keywords:** metabolites, pig, seasonality, seminal plasma, sperm quality

## Abstract

High semen quality is vital for reproductive success in the swine industry; however, seasonal fluctuations often compromise this quality. The molecular mechanism underlying these seasonal effects on semen quality remains largely unclear. This study employed untargeted metabolomic profiling of boar seminal plasma (SP) to identify metabolites and metabolic pathways associated with semen quality during the summer and winter months. Semen samples were collected from mature Duroc boars at a commercial boar stud and classified as Passed or Failed based on motility and morphology. SP from five samples per group was analyzed using ultra-high-performance liquid chromatography–mass spectrometry (UHPLC-MS). In total, 373 metabolites were detected in positive ion mode and 478 in negative ion mode. Several differentially expressed metabolites (DEMs) were identified, including ergothioneine, indole-3-methyl acetate, and avocadyne in the summer, as well as LysoPC, dopamine, and betaine in the winter. These metabolites are associated with key sperm functions, including energy metabolism, antioxidant defense, and capacitation. KEGG pathway analysis indicated enrichment in starch and sucrose metabolism, pyrimidine metabolism, and amino acid metabolism across the seasons. Overall, the results reveal that SP metabolomic profiles vary with the season, thereby influencing semen quality. The identified metabolites may serve as potential biomarkers for assessing semen quality and enhancing reproductive efficiency in swine production.

## Simple Summary

Boar fertility plays a crucial role in pig farming; however, various factors, including seasonal temperature fluctuations, can negatively impact semen quality. This study examines the metabolites present in boar seminal plasma that are associated with sperm quality during both summer and winter seasons. Our findings reveal significant differences between samples collected in the two seasons, with specific metabolites that either protect sperm from oxidative stress or supply energy varying accordingly. Gaining a better understanding of these seasonal fluctuations could enable pig producers to manage boar semen quality more effectively, ultimately leading to more consistent breeding throughout the year.

## 1. Introduction

Boar fertility is a critical factor in enhancing reproductive efficiency and ensuring profitability in pig production. In the swine industry, artificial insemination (AI) is the most extensively utilized breeding method that uses semen from superior males to ensure high fertilization rates and promote genetic progress [1]. High semen quality is essential for the success of AI, which is why semen doses with high sperm motility and normal morphology are used during insemination to improve the genetic quality and reproductive performance of pig herds [2]. Additionally, temperature and seasonality negatively impact semen quality, resulting in significant economic loss for the swine industry. High ambient temperatures during summer have been shown to affect boar sperm quality during spermatogenesis, leading to reduced sperm motility, concentration, and volume [3,4]. Spermatogenesis is highly sensitive to heat stress, and the physiological response to high temperature varies between boar individuals [5,6]. Currently, routine assessments of sperm parameters such as motility, morphology, concentration, and total count are conducted using conventional techniques such as computer-assisted sperm analysis (CASA) to evaluate semen quality. However, these techniques do not provide a comprehensive evaluation of boar fertility or accurately predict the fertility potential of individual boars [7]. Furthermore, semen evaluation cannot identify the underlying factors contributing to subfertility in boars [8]. Consequently, comprehensive techniques are needed to elucidate the molecular mechanisms governing semen quality.

Seminal plasma, the non-cellular liquid portion of semen secreted by the testes, prostate, epididymis, and accessory glands [9], has emerged as a promising biofluid for metabolic analysis of male fertility and semen quality. It contains a diverse range of biomolecules, including proteins, lipids, antioxidants, DNA, RNA, and extracellular vesicles, which play important roles in motility acquisition, capacitation, sperm-oocyte fusion, and immunoregulation of spermatozoa against oxidative stress [10,11,12]. Due to its complex composition, seminal plasma serves as a metabolic support for sperm during critical processes such as sperm maturation, transport in the female reproductive tract, and fertilization [13]. Accordingly, it is a potential source of biomarkers related to semen quality [14,15].

Research is increasingly employing omics techniques such as metabolomics to uncover specific metabolic signatures linked to semen quality [16]. Metabolomics offers a comprehensive approach to identifying biomarkers that could be associated with sperm functionality [17,18], as it enables a thorough characterization of an organism’s phenotype by profiling the metabolic composition of biological fluids [19]. Regarding seminal plasma, several studies have been conducted on livestock species, including pigs. These studies have shown that metabolomic profiling of porcine SP can identify specific metabolites, such as glutamate, D-fructose, betaine, carnitine, and isoleucine, which are associated with energy metabolism, antioxidant capacity, and sperm motility [20,21]. Given that SP provides metabolic support for spermatozoa from ejaculation until fertilization [13,22] and also during seasonal challenges, exploring SP metabolites derived from semen with varying quality statuses could reveal metabolite signatures influencing semen quality.

We propose that metabolic changes related to semen quality remain consistent in boar semen across seasonal variations, suggesting that these intrinsic disruptions may significantly influence fertility. This study aims to employ untargeted metabolomic analysis to identify potential biomarkers in boar seminal plasma that correlate with semen quality. We examine the metabolic pathways that affect sperm function and fertility outcomes, with a specific focus on differences in metabolomic profiles between varying quality statuses (Passed versus Failed) within the same season (winter versus summer) and the intrinsic metabolic factors associated with semen quality, independent of seasonal environmental stressors.

The findings could provide insights into the molecular mechanisms underlying semen quality and inform strategies to enhance reproductive performance in boars.

## 2. Materials and Methods

### 2.1. Animals and Semen Collection

**Figure 1:**
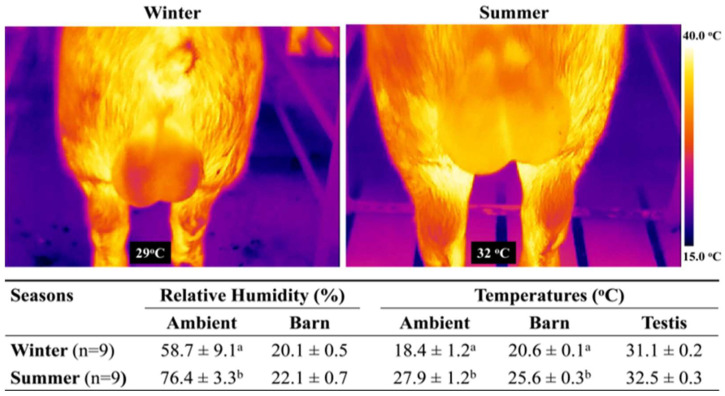
Environmental and boar scrotal temperature monitoring during winter and summer. The Digital Infrared Thermal Imaging (DITI) images illustrate the variations in surface temperatures of boar testes, with scrotal temperatures presented against a dark background. The scale uses pseudo-colors to indicate temperature variations, ranging from the coldest at 15.0 °C to the hottest at 40 °C. Data were collected twice per week over nine weeks (*n* = 9) during winter (February–March) and summer (July–September). Data are presented as mean ± standard error of the mean (sem). Different superscript letters (a, b) within the same column denote significant differences (*p* < 0.05, Student’s *t*-test).

Forty healthy, sexually mature Duroc boars, aged 1.5 to 2 years, were selected as semen donors at Prestage Farms (West Point, MS, USA), a commercial boar stud. The boars were housed in controlled barns with ventilation and temperature monitoring. They were maintained under similar management and nutritional conditions, with ad libitum access to water. Semen ejaculates were collected twice a week, using the gloved-hand method by trained technicians during two seasons: winter (February to March; *n* = 75 single sire semen samples) and summer (July to September; *n* = 83 single sire semen samples). A summary of the ambient and barn environmental factors (temperature and relative humidity) is presented in Figure 1. This figure also includes scrotal temperatures of the boars, which were monitored using a Digital Infrared Thermal Imaging (DITI) camera (FLIR ThermoCAM S60; FLIR Systems, Inc., Boston, MA, USA). DITI images were taken within the barn on randomly selected boars involved in the study, before, during, and after each semen collection. These images were subsequently processed and compiled using the ThermaCAM Researcher Professional v2.7 software.

Aliquots (5 mL) of the semen samples were chilled and immediately transported to our laboratory at Mississippi State University (Starkville, MS, USA) within one hour for processing and further analyses.

### 2.2. Evaluation of Semen Quality

Boar semen (500 µL) was incubated at 37 °C for 15 min. Following incubation, 2 µL of semen was transferred onto pre-warmed, caffeine-free microscope slides (Standard Count 4-chamber Slide Leja^®^, 20 µm, Nieuw Vennep, The Netherlands). Semen analysis was conducted using the CEROS II Computer-Assisted Sperm Analyzer (CASA) from IMV Technologies (Brooklyn Park, MN, USA). A minimum of 1000 spermatozoa were analyzed across four non-consecutive fields, assessing total motility, progressive motility, and morphology. The CASA system was set according to the following cut-off values: 60 frames/s, motility threshold set to 5 µm/s, average path velocity and straightness set to 45 µm/s and 45%, respectively, and cut-offs for slow-moving cells at 20 µm/s and 5 µm/s. The system magnification was set to 1.89×, with a temperature of 37 °C. Semen samples were analyzed in 18 independent collections per season (*n* = 9 weeks) and were classified as “Passed” (high-quality; *n* = 20 boars) if they exhibited more than 70% normal morphology and motility, while samples below this threshold were classified as “Failed” (low-quality; *n* = 20 boars). Table 1 summarizes sample collection and sperm characteristics per replicate (week, with two collections/week) during winter, with a similar approach used for summer samples.

**Figure 2:**
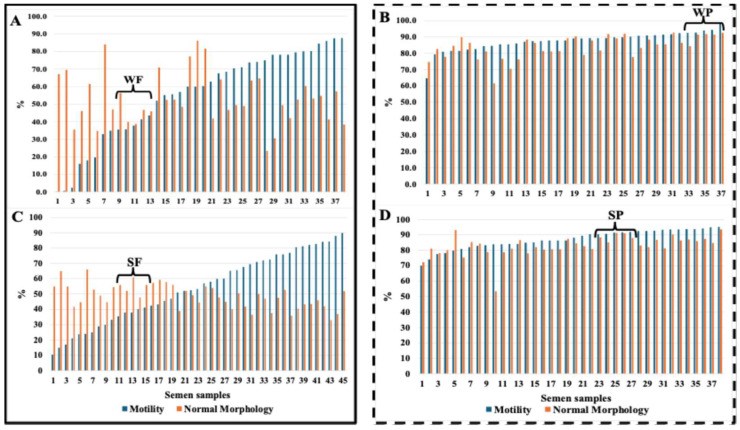
Distribution of individual semen samples based on sperm motility and normal morphology during winter (upper panel—(**A**,**B**)) and summer (lower panel—(**C**,**D**)). The cut-off thresholds for classifying samples as Failed (solid linebox) or Passed (dotted linebox) were set at <70% and ≥70%, respectively, for both sperm motility and normal morphology.

Figure 2 ranks individual semen samples according to sperm motility and normal morphology. It also illustrates the four groups of samples chosen for untargeted metabolomics analysis. These groups consist of five samples, representing a minimum of mean ± 2 standard deviations (SD) of sperm motility from both Passed (mean + 2SD) and Failed (mean − 2SD) semen samples during winter (WP and WF, respectively) and summer (SP and SF, respectively), thereby highlighting the biologically contrasting extremes.

### 2.3. Isolation of Boar Seminal Plasma

Selected semen samples of all groups (*n* = 5 for each WP, WF, SP, and SF group) were centrifuged at 800× *g* for 20 min at room temperature to separate sperm cells. The supernatants were then subjected to a second centrifugation at 2000× *g* for 20 min at 4 °C to remove residual sperm cells and cellular debris. The resulting clarified supernatants, designated as seminal plasma, were stored at −80 °C until metabolomic analysis.

### 2.4. Metabolite Extraction and Untargeted Metabolomics Analysis

#### 2.4.1. Metabolite Extraction

All frozen seminal plasma samples (*n* = 20) were thawed, and 100 μL of each sample was mixed with 300 μL of methanol. The mixture was vortexed for 30 s and sonicated in an ice-water bath for 30 min. Samples were then stored at −20 °C for 1 h, vortexed again for 30 s, and centrifuged at 12,000 rpm for 10 min at 4 °C. Following centrifugation, 200 μL of supernatant was transferred to a new vial and 5 μL of 0.14 mg/mL DL-o-Chlorophenylalanine was added as an internal standard, filtered through a 0.22 μm filter for LC-MS analysis. Quality control (QC) samples were generated by combining equal amounts of extract from each sample and prepared using the same sample preparation procedure to ensure methodology stability.

#### 2.4.2. Untargeted Metabolomics Analysis Using Ultra-Performance Liquid Chromatography–Mass Spectrometry (UPLC-MS)

Chromatographic separation was conducted using the Vanquish Flex UPLC (Ultra-High Performance Liquid Chromatography) in conjunction with a Q Exactive Plus mass spectrometer (Thermo Fisher Scientific, Waltham, MA, USA) equipped with a heated ESI source. The separation was executed on a Waters T3 column (100 × 2.1 mm, 1.8 μm), with the mobile phase consisting of solvent A (0.05% formic acid in water) and solvent B (acetonitrile) utilizing a gradient elution profile: 0–1 min at 5% B; 1–12.5 min from 5% to 95% B; 12.5–13.5 min at 95% B; 13.5–13.6 min from 95% to 5% B; and 13.6–16 min at 5% B. The flow rate of the mobile phase was maintained at 0.3 mL/min, while the column temperature was kept at 40 °C, and the sample manager temperature was set to 4 °C. For mass spectrometry (MS) analysis, Q Exactive Plus was utilized in both positive and negative ion modes via electrospray ionization (ESI). The parameters for the ESI source were set as follows: heater temperature at 300 °C; sheath gas flow rate at 45 arb; auxiliary gas flow rate at 15 arb; sweep gas flow rate at 1 arb; spray Mvoltage set to 3.0 kV for positive ion mode and 3.2 kV for negative ion mode; capillary temperature at 350 °C; and S-Lens RF level adjusted to 30% for positive mode and 60% for negative mode.

#### 2.4.3. Multivariate Statistical Analysis

Raw data were processed and aligned using Compound Discoverer (version 3.0, Thermo Fisher Scientific, Waltham, MA, USA) based on the m/z values and retention times of the ion signals. Ions from both negative (ESI−) and positive (ESI+) were combined and imported into SIMCA-P software (version 14.1, Umetrics, Umeå, Sweden) for multivariate data analysis. Supervised regression modeling (Orthogonal Partial Least Squares Discriminant Analysis or OPLS-DA) was applied to discriminate between sample groups and identify potential biomarkers. The quality of the fitting models was evaluated using R^2^ (R^2^X and R^2^Y) and Q^2^ values, where R^2^ represents the proportion of variance explained by the model, reflecting the model’s quality of fit. R^2^, R^2^X, and R^2^Y represent the proportion of variance explained by the model in the X and Y matrices, respectively, while Q^2^ indicates the model’s predictive ability based on the variance in the data (Supplementary File S1, Table S1). Variable importance in projection (VIP) values of metabolites were calculated using the OPLS-DA model to evaluate the contribution of each variable.

#### 2.4.4. Identification of Potential Biomarkers and Pathway Analysis

Differentially expressed metabolites (DEMs) were selected based on VIP values (>1.5), *t*-test results (*p* < 0.05), and fold change (FC) > 1.50 or <0.66. The chemical structures of metabolites were identified using the Human Metabolome Database (https://www.hmdb.ca/; accessed on 30 August 2024). For validation, DEMs were compared with authentic standards by assessing retention times and MS/MS fragmentation patterns. Metabolites with VIP ≥ 2.0 and *p* < 0.05 were further classified as potential biomarkers. Pathway annotation and enrichment analysis of DEMs were conducted using the Kyoto Encyclopedia of Genes and Genomes (KEGG; https://www.kegg.jp; accessed on 30 August 2024) and MetaboAnalyst (https://www.metaboanalyst.ca; accessed on 30 August 2024). Only well-annotated HMDB compounds (those in pathway libraries and metabolite sets) were mapped. Metabolic pathways with *p*-values < 0.05 were considered significantly enriched.

#### 2.4.5. Cluster Analysis

Mean metabolite values from biological seminal plasma replicates of Summer Failed (SF), Summer Passed (SP), Winter Failed (WF), and Winter Passed (WP) groups were used to calculate metabolite ratios. After log transformation, median-centered ratios were normalized. Hierarchical clustering analysis (HCA) was performed with the complete linkage algorithm from Cluster 3.0 (Stanford University, Stanford, CA, USA) and visualized using Pheatmap 1.0.12 (Raivo Kolde). Ratios from two independent experiments of significant metabolites were included in the HCA. Univariate analyses were performed using volcano plots, including fold change and *t*-tests. Following this, metabolites were grouped into clusters based on their seasonal expression profiles using the Mfuzz Bioconductor package in R (version 4.4.2).

### 2.5. Statistical Analysis

Semen data were analyzed using SPSS for Windows, version 29.0 (SPSS Inc., Chicago, IL, USA). Semen groups (Failed and Passed) were tested for normality using the Shapiro–Wilk’s test, followed by group and seasonality comparisons using the two-way ANOVA. Differences between semen groups (Passed and Failed) and seasons (summer and winter) were considered significant at a *p*-value < 0.05. The results are presented as the mean ± SEM.

## 3. Results

### 3.1. Semen Quality Evaluation

**Figure 3:**
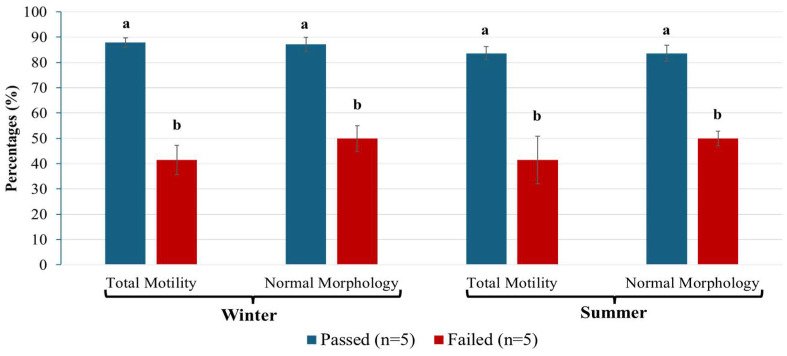
Screening of semen quality parameters during summer and winter semen groups. Total motility and normal morphology between the Passed and Failed semen groups during winter and summer. The data are shown as mean ± SEM, and different letters represent significant differences at *p* < 0.05.

The data are presented in Figure 3. Total motility was significantly higher (*p* < 0.001) in the Passed group compared to the Failed group during both seasons. Specifically, total motility in the Passed group was 87.8% in summer and 83.6% in winter, whereas the Failed group exhibited 41.1% in both seasons. Similarly, normal morphology was significantly greater (*p* < 0.001) in the Passed group, with values of 87.1% in summer and 83.6% in winter, compared to 49.9% in the Failed group during both seasons. No significant seasonal differences were observed within either sperm quality group for total motility (*p* = 0.0714) or normal morphology (*p* = 0.707).

**Figure 4:**
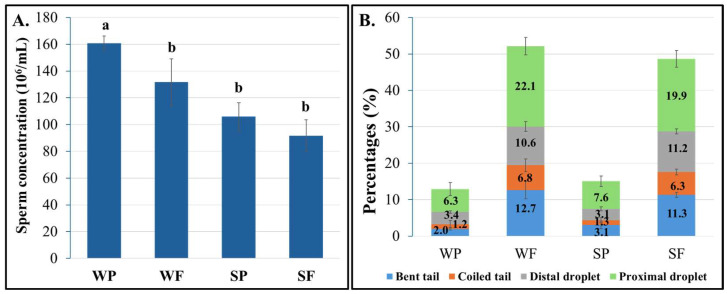
Sperm concentration (**A**) and abnormalities (**B**) of winter and summer semen groups. Experimental groups are WP (Winter Passed), WF (Winter Failed), SP (Summer Passed), and SF (Summer Failed), consisting of 5 boars per group. Different letters (a, b) indicate significant differences between groups (*p* < 0.05).

Sperm concentration was significantly higher in the winter samples (146.3 ± 9.9 vs. 98.8 ± 7.8; *p* < 0.001), with the Winter Passed group showing significantly higher concentrations than all other groups (*p* < 0.05; Figure 4A). Regardless of the seasons, Failed (vs. Passed) samples exhibited significantly higher proportions of sperm abnormalities including bent tails (12.0 ± 1.2% vs. 2.6 ± 0.6%), coiled tails (6.6 ± 0.9% vs. 1.3 ± 0.6%), and distal (10.9 ± 0.7% vs. 3.2 ± 0.3%) and proximal (21 ± 1.6% vs. 7.0 ± 1.1%) droplets in both seasons (*p* < 0.01). Figure 4B illustrates the sperm abnormalities by semen group.

### 3.2. Metabolomic Profiling and Chemical Classification of Boar Seminal Plasma (SP)

**Figure 5:**
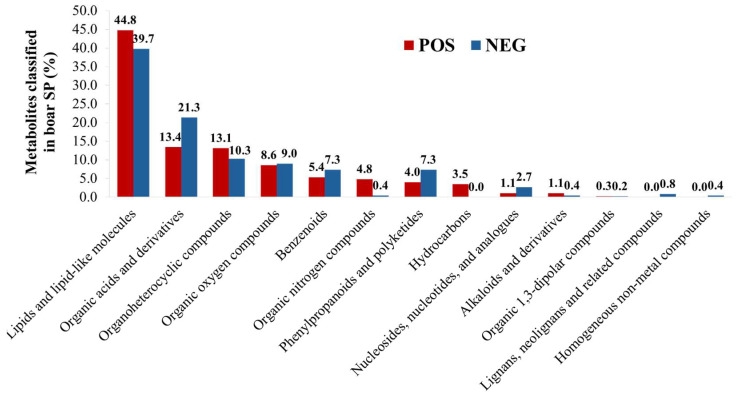
Chemical classification of metabolites in boar seminal plasma (SP) detected in the positive (POS) and negative (NEG) ion modes. Data are a combination of semen samples collected during winter and summer.

Metabolomic analysis of boar SP samples from the winter and summer seasons identified a total of 851 metabolites, with 373 detected in the positive ion mode and 478 in the negative ion mode (Supplementary File S1, Tables S2 and S3). Among these, 32 metabolites (3.8%) were common to both ion modes, resulting in 819 unique metabolites. Figure 5 illustrates that the major chemical classes include lipids and lipid-like molecules, organic acids and derivatives, organoheterocyclic compounds, organic oxygen compounds, and benzenoids, which represented a substantial proportion of the identified compounds, accounting for 84.5% and 86% in positive and negative ion modes, respectively. Notably, certain metabolite classes were unique to specific ionization modes. Hydrocarbons (*n* = 13; e.g., pregeijerene, undecane, aplotaxene, and tetradecane) were exclusively detected in positive ion mode. In contrast, homogeneous non-metal compounds (*n* = 2; sulfate and phosphate) and lignans, neolignans, and related compounds (*n* = 4; enterolactone, 6′-hydroxyenterolactone, enterolactone 3″-sulfate, and enterolactone 3″-glucuronide) were specific to negative ion mode. More details are available in Supplementary File S1, Table S4.

### 3.3. Identification of DEMs in Boar Seminal Plasma Between Summer and Winter Groups

**Figure 6:**
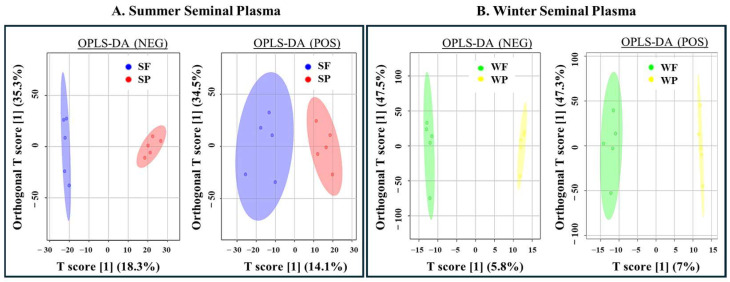
Orthogonal partial least squares-discriminant analysis (OPLS-DA) of boar seminal plasma metabolites during summer (S) and winter (W): (**A**) OPLS-DA score plot in summer samples in the negative and positive ion modes. (**B**) OPLS-DA score plot in winter samples in the negative and positive ion modes. Samples are Summer Failed (SF; blue), Summer Passed (SP; red), Winter Failed (WF; green), and Winter Passed (WP; yellow).

The OPLS-DA was employed to evaluate metabolic changes between the groups based on season (summer and winter) and semen quality status (Passed and Failed). The OPLS-DA score revealed a clear separation between the groups, during summer (SF and SP) and winter (WF and WP), in both positive and negative ion modes, indicating distinct metabolic profiles associated with seasonal variation and semen quality status (Figure 6). In both positive (POS) and negative (NEG) ion modes, the OPLS-DA model showed high R^2^Y values, indicating strong model fit for the summer (0.925 and 0.998) and the winter (0.994 and 0.777) groups. The Q^2^ values were above 0.2 in the summer groups (0.35 and 0.22), but notably lower in the winter groups (−0.105 and −0.846), suggesting weaker model predictability for those samples (Supplementary File S1, Table S1).

**Figure 7:**
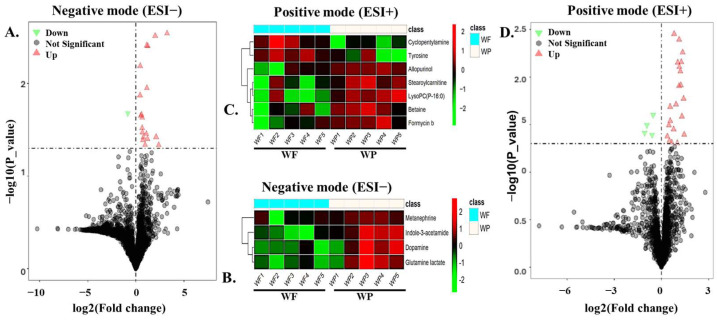
Differential expression and hierarchical cluster analysis of metabolites in boar seminal plasma obtained during winter. Volcano plots (**A**,**D**) and heat maps (**B**,**C**) of DEMs in the negative (ESI−) and positive (ESI+) ion modes. Red represents the up-regulated metabolites, green represents the down-regulated metabolites, and gray represents the metabolites with no difference. Data were obtained from 5 independent replicates of Winter Failed (WF) and Winter Passed (WP).

**Figure 8:**
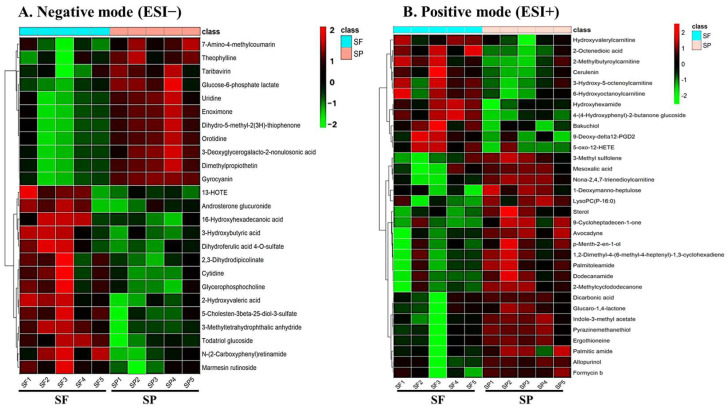
Hierarchical cluster analysis of significant metabolites in boar seminal plasma obtained during summer. Heat maps display the patterns of up-regulated (red) and down-regulated (green) metabolites in the (**A**) negative and (**B**) positive ion modes, comparing samples of Summer Failed (SF) with Summer Passed (SP). Data were obtained from 5 independent replicates for each SF and SP group.

**Figure 9:**
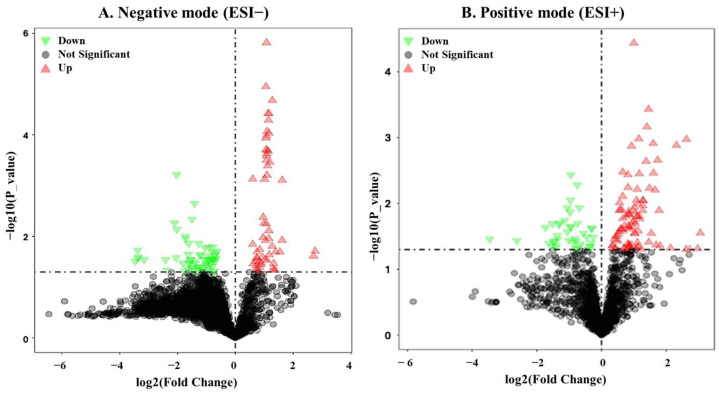
Differential expression of metabolites from boar seminal plasma obtained during summer. Volcano plots of DEMs between Passed and Failed groups in the (**A**) negative (ESI−) and (**B**) positive (ESI+) ion modes. Red represents the up-regulated metabolites, green represents the down-regulated metabolites, and gray represents the metabolites with no difference.

Hierarchical clustering analysis was performed on differentially expressed metabolites (DEMs) with *p* < 0.05 to assess expression patterns in boar seminal plasma samples collected during winter (Figure 7) and summer (Figure 8 and Figure 9). The results were visualized as heatmaps and corresponding volcano plots. The heatmaps revealed distinct clustering and expression profiles between the Passed and Failed groups within each season (WP vs. WF and SP vs. SF), indicating season and semen quality-specific metabolic signatures. The volcano plots further illustrated the distribution, magnitude of fold changes, and significance level of the DEMs between groups.

A total of 68 differentially expressed metabolites (DEMs) were identified as potential biomarkers based on VIP > 1.5 and *p* < 0.05, regardless of sperm quality status (Failed or Passed) and season (summer or winter). The full details of these DEMs are provided in Supplementary File S1 (Tables S5 and S6). In the (ESI) positive ion mode, 32 DEMs were identified in the summer comparison (SF vs. SP) and 7 DEMs in the winter comparison (WP vs. WF). In the (ESI) negative ion mode, 25 and 4 DEMs were identified in the summer and winter group comparisons, respectively. The most significant DEMs across season and sperm quality groups are summarized in Table 2 and Table 3.

Of these significant DEMs, 17 were selected as seasonal candidate biomarkers with potential implications in sperm functionality based on VIP > 2 and *p* < 0.05 (Table 4). Supplementary File S1 (Table S7) provides the lists of additional biomarker candidates indicating allopurinol, formycin b, and avocadyne are common to both seasons.

**Figure 10:**
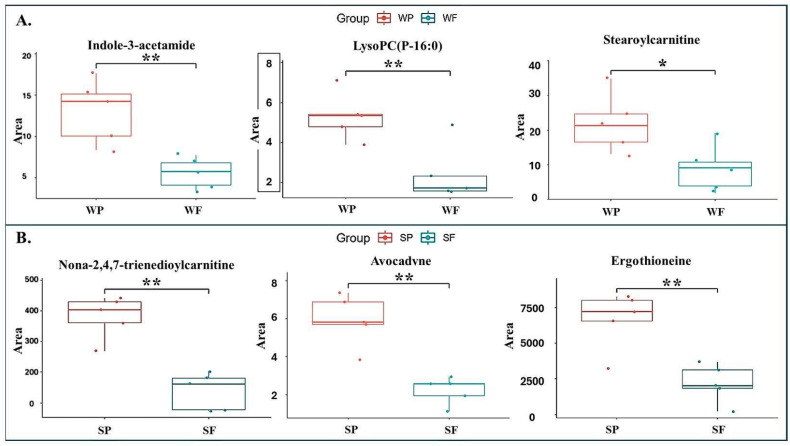
Relative abundance of selected candidate DEMs during winter (**A**) and summer (**B**) boar seminal plasma. The upper panel shows box-and-whisker plots of DEMs for Passed (WP) vs. Failed (WF) samples in winter, while the lower panel displays DEMs for Passed (SP) vs. Failed (SF) samples in summer. Asterisks represent significant difference between groups (* *p* < 0.05; ** *p* < 0.01).

The box plots presented in Figure 10 illustrate the distribution’s shape, central tendency, and variability, corresponding to the maximum and minimum values, lower and upper quartiles, and median of a selection of DEMs (VIP ≥ 2.0 and *p* < 0.05) that may serve as predictors for Passed vs. Failed semen during both winter and summer. 

**Figure 11:**
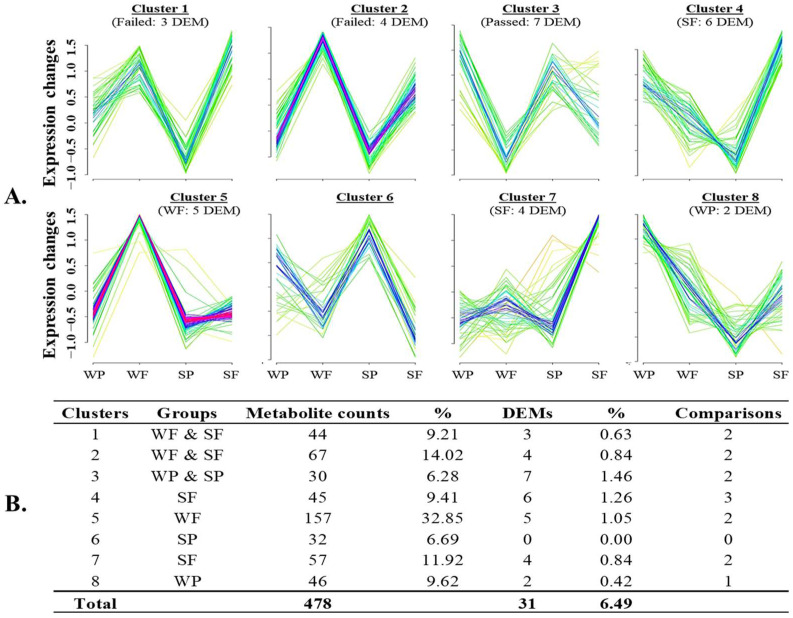
Mfuzz cluster analysis of boar seminal plasma metabolites detected across different seasons using the negative ion mode method. Dynamic patterns of these metabolite clusters (**A**) were linked to various experimental conditions, summarized in Table (**B**), which shows the number and percentage of differentially expressed metabolites (*p* < 0.05) in each cluster. Each line represents an individual metabolite that fits better (red, purple, and blue lines) and less (yellow or green lines) in the cluster’s expression patterns. The experimental groups are designated as WP (Winter Passed), WF (Winter Failed), SP (Summer Passed), and SF (Summer Failed).

**Figure 12:**
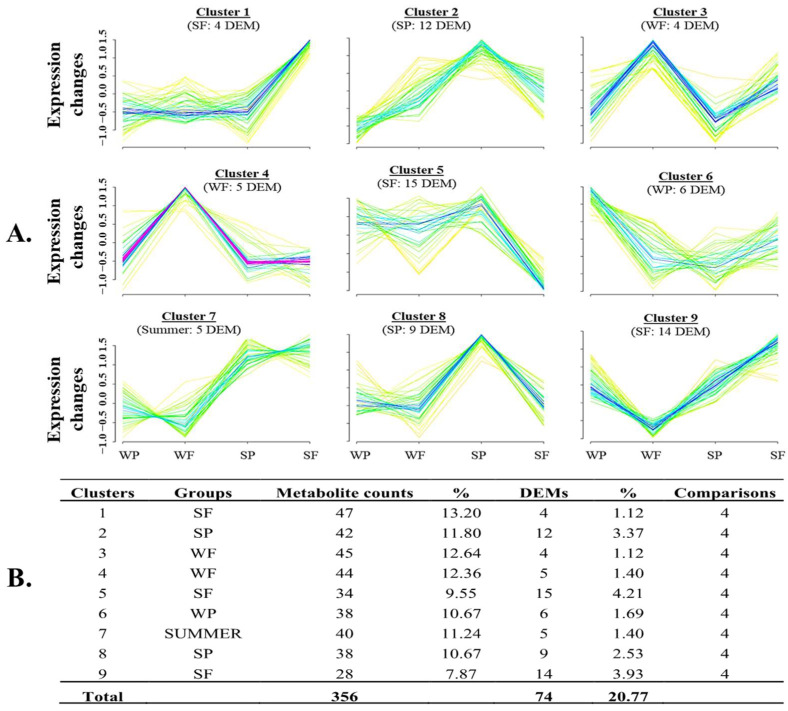
Mfuzz cluster analysis of boar seminal plasma metabolites detected across different seasons using the positive ion mode method. Dynamic patterns of these metabolite clusters (**A**) were linked to various experimental conditions, summarized in Table (**B**), which shows the number and percentage of differentially expressed metabolites (*p* < 0.05) in each cluster. Each line represents an individual metabolite that fits better (red, purple, and blue lines) and less (yellow or green lines) in the cluster’s expression patterns. The experimental groups are designated as WP (Winter Passed), WF (Winter Failed), SP (Summer Passed), and SF (Summer Failed).

Moreover, Mfuzz clustering analysis revealed additional DEMs of interest, demonstrating distinct expression patterns across all four experimental groups (Supplementary File S1, Table S8). In the negative ion mode, eight clusters were observed (Figure 11) and nine in the positive ion mode (Figure 12). In the negative ion mode (Figure 11A), several clusters displayed high metabolite expression patterns that effectively distinguished Failed from Passed samples, regardless of season. For example, clusters 1 and 2 were enriched in the Failed groups and featured decreased levels of 2-hydroxyvaleric acid, androsterone glucuronide, and pantothenic acid. In contrast, cluster 3, which was enriched in the Passed groups, showed increased levels of metabolites such as indole-3-acetamide, dopamine, and glutamine lactate. Additional clusters revealed season-specific distinctions: Failed samples from summer (SF) were characterized by reduced levels of glycerophosphocholine and cytidine (clusters 4 and 7). In contrast, Winter Failed samples (WF) exhibited decreased expression of 13-HOTE and Asparaginyl–Tryptophan (cluster 5).

In the positive ion mode (Figure 12A), distinct clusters also revealed characteristic metabolite expression patterns associated with the summer season, independent of semen quality status. Specific clusters differentiated Summer Failed samples (SF), including clusters 1, 5, and 9, which showed decreased levels of 2-methylbutyroylcarnitine, betaine, linoleic acid, and α-linoleic acid. In contrast, Summer Passed samples (SP) were represented by clusters 2 and 8, characterized by elevated levels of ergothioneine, formycin B, sterol, and tyrosine. For winter samples, Failed groups (WF) were represented by clusters 3 and 4, featuring decreased levels of 6-hydroxyoctanoylcarnitine and 9-deoxy-delta12-PGD2. At the same time, Winter Passed samples (WP) were associated with cluster 6, which included decreased lysoPG (16:0) and hydroxyvalerylcarnitine. Notably, cluster 7 exhibited reduced levels of 24-hydroxytetracosanoic acid, indicative of a general seasonal effect.

### 3.4. Functional Biochemical Pathway Analysis

Metabolic pathway enrichment analysis was performed using the KEGG database to identify pathways associated with differentially expressed metabolites (DEMs) between summer and winter boar seminal plasma samples (Supplementary File S1, Table S9). In the summer group, DEMs were significantly enriched (*p* < 0.05) in six metabolic pathways, including pyrimidine metabolism, glycerophospholipid metabolism, ether lipid metabolism, and butanoate metabolism. In contrast, DEMs from the winter group were enriched in two pathways: tyrosine metabolism and glycine, serine, and threonine metabolism (*p* < 0.05). Notably, starch and sucrose metabolism were significantly enriched in both seasonal groups (*p* < 0.05) (Table 5).

## 4. Discussion

Sperm motility and morphology are key indicators of semen quality and boar fertility. This study employed untargeted metabolomic analysis to identify potential biomarkers associated with semen quality and molecular pathways that affect sperm function. Semen classified as “Passed” demonstrated higher motility and a greater proportion of morphologically normal sperm compared to “Failed” samples, regardless of season. Therefore, understanding the underlying molecular mechanisms at the semen level, which are undoubtedly linked to individual differences among boars, can permit the prediction or significant enhancement of the reproductive performance of swine herds.

Various factors, including thermoregulation, cause sire-to-sire variations in male fertility outcomes. Seasonal temperature and humidity differences between winter and summer were monitored with routine methods, alongside digital infrared thermography, an accurate and non-invasive imaging technique, to evaluate testicular conditions. Our findings revealed a high prevalence of abnormal spermatozoa in Failed semen and a notable decrease in sperm concentration during summer, regardless of semen quality (Passed or Failed). These findings align with previous research in ruminants, where even moderate increases in scrotal temperature negatively impact sperm production, motility, and viability [23,24,25]. The increased proportion of abnormal spermatozoa in Failed semen (3–4 times more than in Passed semen) was linked to a higher incidence of secondary sperm defects affecting motility. Additionally, the rise in primary defects (approximately 1.5 times higher) suggests a lower fertility potential of Failed semen. Further investigation into the primary defects, particularly acrosome and head abnormalities, could provide valuable insights into reducing rejection rates in commercial boar studs during hotter months [26,27].

Interestingly, the Winter Passed semen group, which exhibited the highest sperm concentrations and the lowest sperm defective rate at 12.9%, may serve as a useful benchmark for future therapeutic studies. In contrast, the Summer Passed semen had a slightly elevated abnormality rate (15.1%), likely due to minor increases in testicular temperature leading to abnormal sperm production and lower sperm concentrations [28]. Hence, other disturbances in the testes may exist, as moderate testicular temperature increases can elevate metabolism and oxygen demand, causing hypoxia and altered spermatogenesis [29]. In this context, seminal plasma, a fluid rich in bioactive molecules, could be a valuable source for detecting molecular changes that reflect each boar’s unique response to thermal stress.

Seminal plasma plays a crucial role in various aspects of sperm function, providing protective and nutritive support essential for sperm maturation, energy metabolism, motility, capacitation, and fertilization [30,31,32]. As spermatozoa traverse the epididymis, they are exposed to metabolites, which can trigger metabolic reactions and mechanisms that lead to downstream changes in gene expression. Metabolomic analysis is therefore a powerful tool for identifying biomarkers associated with sperm phenotypes and for elucidating the metabolomic pathways that impact male fertility [33,34,35].

The predominant chemical classes of metabolites identified in boar SP included lipids and lipid-like molecules, followed by organic acids and derivatives, organoheterocyclic compounds, and organic oxygen compounds, consistent with previous studies on boar semen, boar SP, and bull sperm metabolites [36,37,38]. Lipids and lipid-like molecules contribute to structural integrity and membrane stability, thereby influencing sperm motility, capacitation, and viability [39,40]. Organic acids, which are byproducts of amino acid and fatty acid catabolism, serve as energy substrates that replenish the tricarboxylic acid (TCA) cycle [41]. The abundance of these metabolites, alongside lipids, suggests a high level of energy metabolism in boar spermatozoa to sustain motility, survival, and fertilization capacity [40,42]. The differential sensitivity of positive and negative ion modes during metabolomic analysis also highlighted variable detection efficiency across less abundant metabolite classes, such as hydrocarbons, lignans, neolignans, and related compounds, as well as homogeneous non-metal compounds.

In this study, OPLS-DA analysis revealed distinct metabolic profiles between summer and winter boar seminal plasma samples. Among the most abundant DEMs in the summer were gyrocyanin, nona-2,4,7-trienedioylcarnitine, avocadyne, indole-3-methyl acetate, ergothioneine, and palmitoleamide. Avocadyne, a long-chain fatty alcohol (polyhydroxylated fatty alcohols or PFAs), has been shown to modulate mitochondrial metabolism, which selectively suppresses fatty acid oxidation (FAO) in leukemia cells [43]. While its role in sperm function is not explicitly detailed in the current scientific literature, it could play a crucial role in minimizing lipid peroxidation, preserving membrane integrity, and shifting energy metabolism towards glycolysis, a more efficient pathway [44,45].

Similarly, ergothioneine, a naturally occurring antioxidant, protects sperm against reactive oxygen species (ROS) through the mitochondrial respiratory chain [46,47]. In comparison to other antioxidants such as trolox and glutathione, ergothioneine has been shown to have the highest antioxidant activity [48] and higher doses of ergothioneine improved post-thaw quality and membrane integrity in ram [49] and rooster semen [50]. High ergothioneine levels were also found in the seminal plasma of boars [51], and a recent study by Guo et al. [47] indicated that ergothioneine reduces oxidative damage to sperm by enhancing the total antioxidant activity. Therefore, ergothioneine’s elevated expression in the summer boar seminal plasma may help reduce ROS levels and cell death induced by hydrogen peroxide (H2O2), which is intensified by higher temperatures, thereby enhancing sperm membrane stability and motility [50,51]. Moreover, palmitoleamide, a fatty amide, may play a role in preserving sperm viability during cryopreservation by reducing osmotic stress on sperm cells due to its lower molecular weight in comparison to glycerol [52]. Indole-3-methyl acetate is an esterified derivative of indole-3-acetic acid, a well-known plant hormone (auxin) that has also been identified in mammalian and microbial metabolic pathways, particularly those related to tryptophan metabolism and indole derivatives [53]. Differential abundance of indole-3-methyl acetate may reflect shifts in seminal microbial activity or microbiota dysbiosis, both of which can influence sperm quality.

In contrast, the winter boar SP samples were enriched with indole-3-acetamide, LysoPC (P-16:0), stearoylcarnitine, dopamine, glutamine lactate, betaine, and tyrosine. LysoPC, a lysophosphatidylcholine and component of sperm membranes, is known to induce sperm capacitation and has been widely used in murine species [54]. It also influences mitochondrial ROS production through a calcium-dependent mechanism [55]. Based on these findings, we hypothesize that LysoPC may facilitate sperm functionality by promoting cholesterol efflux and hyperactivation in highly motile sperm [56]. Additionally, LysoPC has been found at higher levels in the sperm of high-fertility bulls, where it plays a role in improving sperm motility and acrosome reaction, as well as contributing to membrane fusion events during fertilization [57,58]. Dopamine, a neurotransmitter in the mammalian central nervous system, also participates in sperm physiology [59,60]. High concentrations of dopamine were reported to increase human sperm motility [60], and boar sperm incubated with 100 nM dopamine displayed a significant decrease in dead sperm, suggesting a protective effect of dopamine treatment on sperm viability [61]. This finding reveals seasonal variations in dopamine, aligning with studies that show higher levels in winter [59,60]. This suggests a complex interaction between environmental factors and neurochemistry that affects mood and behavior year-round, contributing to high-quality semen.

Glutamine lactate was also among the metabolites highly expressed in the winter samples. They are two key metabolic substrates that provide energy and support cellular metabolism. Lactate facilitates glutamine uptake, and glutamine metabolism can produce lactate, which can be converted to pyruvate, thereby fueling the Krebs cycle [62]. Hence, the glutamine–lactate interaction may be crucial in sperm metabolism, affecting sperm motility. Furthermore, glutamine has been used as a cryoprotectant during sperm freezing in various species such as rabbits [63], bulls [64], and boars [65]. It is one of the most abundant free amino acids in the body and plays a role in boar sperm development by regulating inflammatory responses, maintaining cell integrity, and protecting cells against oxidative stress by enhancing glutathione (GSH) synthesis [63,66,67]. Based on this, we also speculate that glutamine lactate may contribute to the antioxidant defense mechanism, thereby protecting spermatozoa from oxidative stress.

Furthermore, betaine, a potential biomarker for semen quality during winter, ranked among the most abundant metabolites. This naturally occurring compound, found in plants, animals, and microorganisms, has been utilized as a feed additive in animal husbandry to improve sperm quality and fertility [21,68]. Betaine also acts as an antioxidant, enhancing sperm motility as evidenced by its protective effects on boar sperm when added to semen extenders stored at 17 °C [21,69]. Thus, the presence of betaine in winter boar semen suggests its significant role in enhancing semen quality in boars.

Interestingly, allopurinol and formycin B metabolites were differentially expressed across both seasons. Allopurinol is a xanthine oxidase inhibitor that improves sperm function by reducing the damage to the germ epithelium and apoptosis [70]. Excessive ROS can lead to oxidative stress, which adversely affects sperm quality. The ability of allopurinol to inhibit xanthine oxidase activity suggests its protective effects against oxidative stress [71]. In contrast, formycin B is a naturally occurring isomer of the adenosine nucleoside that features a pyrazole moiety with antimicrobial and antitumor properties by inhibiting cell proliferation [72,73]. Furthermore, correlation analysis between plasma differential metabolites, reproductive hormones, and antioxidant indicators of Hu sheep supplemented with L-citrulline revealed a negative association with formycin B, indicating immune modulation and oxidative response [74]. Overall, the relationship between these DEMs, seasonality, and semen quality underscores their potential as candidate biomarkers for sperm quality.

The differential expression of these metabolites during the winter season likely reflects physiological adaptations of boars to seasonal stressors such as cold temperatures, altered metabolism, and changes in SP biochemical composition, all of which can influence sperm quality, fertility, and overall sperm physiology [75,76]. Winter is generally favorable for pig breeding due to moderate temperatures and reduced heat stress, which enhances sperm concentration and quality compared to the summer season [3,77,78]. Notably, cyclopentylamine and tyrosine were elevated in the Failed samples. Cyclopentylamine, a cyclic amine and chemokine receptor antagonist, may be associated with immune cell migration and altered nitrogen metabolism [79], while tyrosine, a precursor of catecholamines and neurotransmitters such as norepinephrine and dopamine, plays a key role in protein phosphorylation, a critical process for sperm capacitation [80,81,82]. Elevated tyrosine levels may reflect dysregulated capacitation processes in subfertile ejaculates and excessive protein turnover in poor-quality semen [83]. These metabolites could serve as potential indicators of compromised semen quality.

The KEGG enrichment analysis further revealed that summer samples were significantly enriched in various metabolic pathways related to energy production, nucleotide synthesis, and regulation of sperm quality [84]. For example, sucrose, a primary energy source in mammals, is broken down to glucose and fructose [85]. Fructose, found abundantly in boar seminal plasma, serves as the main energy substrate for glycolysis, supporting sperm motility, capacitation, and fertilization [86,87]. Starch and sucrose metabolism in this study was associated with 3-deoxyglycerogalacto-2-nonulosonic acid and glucaro-1,4-lactone, playing a role during sperm capacitation. The notable enrichment of pyrimidine metabolism during summer sample (SF vs. SP) comparisons is crucial for sperm maturation, morphology, and motility. In the SP group, cytidine levels decreased, potentially resulting in reduced 5-methylcytidine, which is associated with improved testicular morphology and sperm quality (higher sperm concentration and enhanced motility) in mice [88]. Conversely, uridine levels increased and may support motility but could suppress early capacitation [89,90]. Glycerophosphocholine, a derivative of unsaturated fatty acids and vital for sperm viability and motility [58], was enriched in both glycerophospholipid and ether lipid metabolism pathways. Glycerophospholipid metabolism contributes to sperm motility [91] and has been shown to reduce oxidative stress, thereby improving sperm quality in stallions [92] and in post-thaw human sperm [93]. Butanoate metabolism, associated with 3-hydroxybutyric acid, was also enriched, critical for energy production in low glucose conditions and essential for maintaining sperm motility [94,95].

In winter samples, tyrosine metabolism showed enrichment, particularly in the WP group, as indicated by elevated dopamine levels. These levels can enhance or inhibit sperm function, depending on the concentration. At low concentrations, dopamine enhances motility and viability, while exhibiting inhibitory effects at high concentrations [96,97]. Although increased metanephrine levels were also observed in the WP group, their role in male fertility remains unclear [98]. Tyrosine metabolism further contributes to sperm motility and capacitation through tyrosine phosphorylation [83,99], as well as to seminal plasma antioxidant [100], playing a possible role in regulating ROS levels and enhancing boar sperm cryotolerance. Glycine, serine, and threonine metabolism were also enriched in winter samples, with potential implications in sperm maturation and capacitation [101]. Glycine has been shown to enhance motility and to initiate acrosome reaction in both human and porcine sperm [102]. Glycine supplementation significantly improved post-thaw sperm quality in monkeys [103], while serine and threonine support structural integrity in sperm by linking doublet microtubules to outer dense fibers [104]. Furthermore, betaine, a metabolite linked to this pathway and found at higher levels in the WP group, is known to positively affect semen quality and sperm preservation [105,106]. Although some metabolic shifts remain speculative regarding direct causation, they align with known sperm biology, suggesting that disruptions in energy metabolism, oxidative stress management, and lipid remodeling are key to understanding the differences between Passed (fertile) and Failed (sub-fertile) ejaculates.

In summary, these candidate biomarkers show promise for improving semen quality and enhancing boar reproductive performance across seasonal variations. However, limitations such as a relatively small sample size and intrinsic variability among boars may contribute to some of the observed metabolic differences. Lastly, as this study focused solely on Duroc boars within a single commercial population, the generalizability of the findings to other breeds, management systems, or geographic locations may be limited. Future research should focus on validating these DEMs in larger, more diverse populations and across multiple breeds and environments to assess their applicability within the swine industry.

## 5. Conclusions

This study highlights the significant seasonal variations in the seminal plasma metabolomic profiles of boars, revealing distinct metabolites and metabolic pathways associated with semen quality in both summer and winter. Key metabolites such as ergothioneine, avocadyne, LysoPC, allopurinol, formycin B, and betaine were identified to play essential roles in modulating sperm function, energy metabolism, and antioxidant defense mechanisms in response to environmental changes. Moreover, the seasonal enrichment of specific metabolic pathways further emphasizes their importance in supporting sperm motility, viability, and fertilization capacity. These findings offer valuable insights that could contribute to further research avenues for the development of targeted strategies to improve fertility outcomes and reproductive performance in swine production systems.

## Figures and Tables

**Table 1:** Classification of winter semen based on total sperm motility or morphology percentages.

Replicates (Weeks)	Semen Samples	Failed (Mean ± Sem; %)		Passed (Mean ± Sem; %)	
	*N*	Motility	Morphology	Motility	Morphology
I	6	86 ± 1	56 ±1	90 ± 2	82 ± 3
II	16	47 ± 3	57 ± 2	90 ± 1	83 ± 2
III	7	64 ± 6	49 ± 1	88 ± 1	87 ± 1
IV	6	45 ± 7	61 ± 6	87 ± 5	87 ± 3
V	8	63 ± 5	41 ± 5	88 ± 1	85 ± 1
VI	11	43 ± 4	52 ± 2	90 ± 0.2	71 ± 4
VII	7	75 ± 2	46 ± 1	80 ± 3	81 ± 1
VIII	6	n.a.	n.a.	89 ± 1	73 ± 6
IX	8	63 ± 4	58 ± 3	86 ± 1	83 ± 2
Global averages		55 ± 0.7	53 ± 0.4	88 ± 0.2	84 ± 0.2
		*N* = 38		*N* = 37	

The designation “n.a.” refers to samples that were not available. Semen was collected twice a week, for a total of 18 collections. A similar methodology was applied to the summer samples.

**Table 2:** DEMs between Passed and Failed boar seminal plasma groups during winter.

HMDB ID	Compound Name	Chemical Formula	FC (WF/WP)	*p*-Value	VIP	ESI
HMDB0029739	Indole-3-acetamide	C_10_H_10_N_2_O	2.352	0.004	2.639	Negative
HMDB0010407	LysoPC(P-16:0)	C_24_H_50_NO_6_P	2.203	0.008	2.378	Positive
HMDB0014581	Allopurinol	C_5_H_4_N_4_O	2.301	0.009	2.350	Positive
HMDB0252811	Glutamine lactate	C_8_H_14_N_2_O_6_	1.573	0.023	1.899	Negative
HMDB0000848	Stearoylcarnitine	C_25_H_49_NO_4_	2.469	0.026	2.048	Positive
HMDB0000073	Dopamine	C_8_H_11_NO_2_	1.556	0.030	1.939	Negative
HMDB0250671	Cyclopentylamine	C_5_H_11_N	0.528	0.032	2.511	Positive
HMDB0000043	Betaine	C_5_H_12_NO_2_	1.391	0.032	1.845	Positive
HMDB0252449	Formycin b	C_10_H_12_N_4_O_5_	1.715	0.036	2.029	Positive
HMDB0004063	Metanephrine	C_10_H_15_NO_3_	1.694	0.038	1.805	Negative
HMDB0000158	Tyrosine	C_9_H_11_NO_3_	0.477	0.039	2.284	Positive

**Table 3:** The topmost DEMs between Passed and Failed boar seminal plasma groups during summer.

HMDB ID	Compound Name	Chemical Formula	FC (WF/WP)	*p*-Value	VIP	ESI
HMDB0034126	Gyrocyanin	C_17_H_12_O_5_	2.1	1.52 × 10^−6^	2.0	Negative
HMDB0251405	Dimethylpropiothetin	C_5_H_10_O_2_S	2.2	3.81 × 10^−5^	1.9	Negative
HMDB0000788	Orotidine	C_10_H_12_N_2_O_8_	2.1	1.97 × 10^−4^	1.8	Negative
HMDB0040150	Dihydro-5-methyl-2(3H)-thiophenone	C_5_H_8_OS	2.1	1.98 × 10^−4^	1.8	Negative
HMDB0015599	Enoximone	C_12_H_12_N_2_O_2_S	2.1	2.60 × 10^−4^	1.8	Negative
HMDB0000425	3-Deoxyglycerogalacto-2-nonulosonic acid	C_9_H_16_O_9_	2.1	3.16 × 10^−4^	1.8	Negative
HMDB0241782	Nona-2,4,7-trienedioylcarnitine	C16H_23_NO_6_	2.7	3.70 × 10^−4^	2.3	Positive
HMDB0035473	Avocadyne	C_17_H_32_O_3_	2.6	6.88 × 10^−4^	2.4	Positive
HMDB0000296	Uridine	C_9_H_12_N_2_O_6_	2.0	7.50 × 10^−4^	1.7	Negative
HMDB0029738	Indole-3-methyl acetate	C_11_H_11_NO_2_	6.2	1.06 × 10^−3^	2.3	Positive
HMDB0036186	Pyrazinemethanethiol	C_5_H_6_N_2_S	3.3	2.20 × 10^−3^	2.3	Positive
HMDB0003045	Ergothioneine	C_9_H_15_N_3_O_2_S	3.0	3.48 × 10^−3^	2.2	Positive
HMDB0245936	3-Methyltetrahydrophthalic anhydride	C_9_H_10_O_3_	0.2	5.42 × 10^−3^	2.0	Negative
HMDB0247230	7-Amino-4-methylcoumarin	C_10_H_9_NO_2_	2.2	1.11 × 10^−2^	1.5	Negative
HMDB0259391	5-Cholesten-3beta-25-diol-3-sulfate	C_27_H_46_O_5_S	0.3	1.11 × 10^−2^	1.8	Negative
HMDB0041404	2-Methylcyclododecanone	C_13_H_24_O	1.5	1.27 × 10^−2^	2.2	Positive
HMDB0256086	Palmitoleamide	C_16_H_31_NO	1.5	1.27 × 10^−2^	2.3	Positive
HMDB0252449	Formycin b	C_10_H_12_N_4_O_5_	2.1	1.38 × 10^−2^	2.0	Positive
HMDB0001889	Theophylline	C_7_H_8_N_4_O_2_	1.5	1.44 × 10^−2^	1.8	Negative
HMDB0014581	Allopurinol	C_5_H_4_N_4_O	1.8	1.48 × 10^−2^	2.1	Positive

**Table 4:** Selected DEMs during winter and summer as seasonal biomarker candidates.

HMDB ID	Compound Name	Chemical Formula	FC (WF/WP)	*p*-Value	VIP	ESI	Season
HMDB0029739	Indole-3-acetamide	C_10_H_10_N_2_O	2.4	0.004	2.6	Negative	Winter
HMDB0010407	LysoPC(P-16:0)	C_24_H_50_NO_6_P	2.2	0.008	2.4	Positive	Winter
HMDB0014581	Allopurinol	C_5_H_4_N_4_O	2.3	0.009	2.4	Positive	Winter
HMDB0000848	Stearoylcarnitine	C_25_H_49_NO_4_	2.5	0.026	2.0	Positive	Winter
HMDB0252449	Formycin b	C_10_H_12_N_4_O_5_	1.7	0.036	2.0	Positive	Winter
HMDB0000158	Tyrosine	C9H_11_NO_3_	0.5	0.039	2.3	Positive	Winter
HMDB0241782	Nona-2,4,7-trienedioylcarnitine	C_16_H_23_NO_6_	2.74	0.0004	2.3	Positive	Summer
HMDB0035473	Avocadyne	C_17_H_32_O_3_	2.65	0.0007	2.4	Positive	Summer
HMDB0029738	Indole-3-methyl acetate	C_11_H_11_NO_2_	6.18	0.0011	2.3	Positive	Summer
HMDB0003045	Ergothioneine	C_9_H_15_N_3_O2S	3.05	0.0035	2.2	Positive	Summer
HMDB0256086	Palmitoleamide	C_16_H_31_NO	1.46	0.0127	2.3	Positive	Summer
HMDB0252449	Formycin b	C10H_12_N_4_O_5_	2.08	0.0138	2.0	Positive	Summer
HMDB0014581	Allopurinol	C_5_H_4_N_4_O	1.81	0.0148	2.1	Positive	Summer
HMDB0243861	1-Deoxymanno-heptulose	C_7_H_14_O_6_	2.15	0.0158	2.1	Positive	Summer
HMDB0060512	Sterol	C_17_H_28_O	1.51	0.0245	2.0	Positive	Summer
HMDB0000378	2-Methylbutyroylcarnitine	C_12_H_23_NO_4_	0.32	0.0404	2.2	Positive	Summer
HMDB0041862	Glucaro-1,4-lactone	C_6_H_8_O_7_	1.74	0.0409	2.0	Positive	Summer

**Table 5:** Metabolic pathway analysis of differentially expressed metabolites in boar seminal plasma samples from the summer and winter groups in negative and positive ion modes (ESI).

Season	Pathway Name	Selected Metabolites in Pathway	*p*-Value	ESI
Summer	Starch and sucrose metabolism	Glucaro-1,4-lactone, 3-Deoxyglycerogalacto-2-nonulosonic acid	0.0011	Both
Summer	Pyrimidine metabolism	Uridine, Cytidine	0.0021	Negative
Summer	Glycerophospholipid metabolism	Glycerophosphocholine	0.0158	Negative
Summer	Ether lipid metabolism	Glycerophosphocholine	0.0158	Negative
Summer	Butanoate metabolism	3-Hydroxybutyric acid	0.0216	Negative
Winter	Tyrosine metabolism	Tyrosine, Dopamine, Metanephrine	0.0311	Both
Winter	Glycine, serine, and threonine metabolism	Betaine	0.0468	Positive

## Data Availability

All data are provided in the manuscript and the Supplementary File.

## Supplementary Materials

The following supporting information can be downloaded at: https://www.mdpi.com/article/10.3390/biology14080995/s1, Supplementary File S1: Metabolomes of boar semen during winter and summer. Table S1: Model parameters. Table S2: Winter metabolome. Table S3: Summer metabolome. Table S4: Chemical classifications. Table S5: DEMs during winter. Table S6: DEMs during Summer. Table S7: Biomarker candidates. Table S8: Mfuzz Clustering. Table S9: KEGG Pathways.

## Abbreviations

The following abbreviations are used in this manuscript:

ANOVA	Analysis of variance
AI	Artificial insemination
CASA	Computer-assisted sperm analysis
DNA	Deoxyribonucleic acid
DEMs	Differentially expressed metabolites
DITI	Digital Infrared Thermal Imaging
ESI	Electrospray ionization
FAO	Fatty acid oxidation
FC	Fold change
GSH	Glutathione synthesis
HCA	Hierarchical clustering analysis
HMDB	Human metabolome database
KEGG	Kyoto encyclopedia of genes and genomes
NEG	Negative
OPLS-DA	Orthogonal partial least squares discriminant analysis
PLS-DA	Partial least squares discriminant analysis
PFAs	Polyhydroxylated fatty alcohols
POS	Positive
PCA	Principal component analysis
QC	Quality control
ROS	Reactive oxygen species
RNA	Ribonucleic acid
SP	Seminal plasma
SEM	Standard error of mean
SPSS	Statistical package for the social sciences
SF	Summer failed
SP	Summer passed
TCA	Tricarboxylic acid cycle
UPLC-MS	Ultra-performance liquid chromatography-mass spectrometry
VIP	Variable importance in projection
WF	Winter failed
WP	Winter passed
